# Prediction of the as Low as Diagnostically Acceptable CT Dose for Identification of the Inferior Alveolar Canal Using 3D Convolutional Neural Networks with Multi-Balancing Strategies

**DOI:** 10.3390/diagnostics13071220

**Published:** 2023-03-23

**Authors:** Asma’a Al-Ekrish, Syed Azhar Hussain, Hebah ElGibreen, Rana Almurshed, Luluah Alhusain, Romed Hörmann, Gerlig Widmann

**Affiliations:** 1 Department of Oral Medicine and Diagnostic Sciences, College of Dentistry, King Saud University, Riyadh 11545, Saudi Arabia; 2 Department of Computer Science, Munster Technological University, Rossa Ave, Bishopstown, T12 P928 Cork, Ireland; 3 Information Technology Department, College of Computer and Information Sciences, King Saud University, Riyadh 11451, Saudi Arabia; 4 Artificial Intelligence Center of Advanced Studies (Thakaa), King Saud University, Riyadh 145111, Saudi Arabia; 5 Division of Clinical and Functional Anatomy, Medical University of Innsbruck, Müllerstrasse 59, 6020 Innsbruck, Austria; 6 Department of Radiology, Medical University of Innsbruck, Anichstr. 35, 6020 Innsbruck, Austria

**Keywords:** 3D imaging, CT scans, as low as diagnostically acceptable dosage, balancing strategies, convolutional neural network

## Abstract

Ionizing radiation is necessary for diagnostic imaging and deciding the right radiation dose is extremely critical to obtain a decent quality image. However, increasing the dosage to improve the image quality has risks due to the potential harm from ionizing radiation. Thus, finding the optimal as low as diagnostically acceptable (ALADA) dosage is an open research problem that has yet to be tackled using artificial intelligence (AI) methods. This paper proposes a new multi-balancing 3D convolutional neural network methodology to build 3D multidetector computed tomography (MDCT) datasets and develop a 3D classifier model that can work properly with 3D CT scan images and balance itself over the heavy unbalanced multi-classes. The proposed models were exhaustively investigated through eighteen empirical experiments and three re-runs for clinical expert examination. As a result, it was possible to confirm that the proposed models improved the performance by an accuracy of 5% to 10% when compared to the baseline method. Furthermore, the resulting models were found to be consistent, and thus possibly applicable to different MDCT examinations and reconstruction techniques. The outcome of this paper can help radiologists to predict the suitability of CT dosages across different CT hardware devices and reconstruction algorithms. Moreover, the developed model is suitable for clinical application where the right dose needs to be predicted from numerous MDCT examinations using a certain MDCT device and reconstruction technique.

## 1. Introduction

In medical diagnostic imaging, ionizing radiation is frequently used [1]. Ionizing radiation may be potentially harmful to patients, with the risk of harm rising with an increasing radiation dose [2]. However, decreasing the radiation dose may reduce several parameters of image quality [3,4]. Therefore, the radiation dose imparted by an examination should be optimized to be as low as diagnostically acceptable (ALADA), meaning that it should be the lowest dose that will still allow for acceptable diagnostic accuracy [5]. The need for dose optimization is especially acute in imaging with multidetector computed tomography (MDCT) because of the relatively higher dose and increasing usage of this modality [1].

To produce an MDCT image, a MDCT scanner exposes the patient to a certain amount of radiation and then acquires the raw imaging data in the form of electrical signals. Reconstruction algorithms, or reconstruction techniques, then process the acquired signals to produce a visible image [6]. Using different MDCT machines or different reconstruction techniques or different radiation doses may affect the quality of the resultant images, and hence the diagnostic accuracy may be variable. Furthermore, different diagnostic tasks have different quality requirements, and hence different acceptable MDCT dose thresholds [7].

Previous cadaveric studies have been conducted to determine what the ALADA dose is for the identification of the position of the inferior alveolar canal (IAC) for various combinations of MDCT scanners and reconstruction techniques [8,9]. The reference standard for dose optimization or determining the ALADA dose for any diagnostic task is to acquire multiple images with various combinations of imaging parameters and resultant radiation doses, and to assess the diagnostic accuracy of examiners using such images. However, this method is conducted manually, which is time consuming, and is influenced by numerous variables that are frequently changed as well as the constant introduction of newer imaging hardware and reconstruction algorithms [7]. Therefore, the ALADA doses determined using the standard method might not be generalizable to different imaging devices or even the same devices, if different reconstruction algorithms are used to process the images. Therefore, an autonomous technique is needed to determine the ALADA dose that may be applicable to any combination of imaging devices and processing algorithms.

Deep learning (DL) is one technique that has been used for the classification of MDCT images [10]. However, the current solutions proposed in the literature for the classification of MDCT images were not developed for prediction of ALADA doses [10,11]. Furthermore, scalability is a concern, because the current models are data dependent and mostly target a certain device with a specific reconstruction method. Moreover, most of the developed models are for use with 2D images, and neglect the volumetric information that 3D images provide. Handling 2D images that are generated by MDCT scanners as separate slices discards the depth of volumetric information and causes the diagnostic accuracy to vary, resulting in a poor performance [10]. Alternatively, reconstructing 3D images from the slices can improve the accuracy but may introduce other limitations such as the variable volume size and require increased computational memory [11]. Additionally, ALADA studies usually have a relatively lower number of images acquired with the ALADA dose compared to non-ALADA images. This difference in the number of cases within the various classifications causes the collected datasets to be highly unbalanced and is expected to introduce critical issues in the performance of existing models. Therefore, a robust method is needed that can train a model, using an unbalanced training set, to analyze 3D datasets and identify which images were acquired with the ALADA dose.

This paper proposes a novel classification method that handles unbalanced datasets of 3D images, and leverages all the information in the images. Its contribution can be summarized as follows:

1. Using MDCT data from previous cadaveric studies [8,9], a new 3D MDCT dataset was built from each existing folder of multiple 2D images [8,9]. The datasets used were comprised of MDCT images acquired with different hardware devices and reconstruction algorithms in order to scale the proposed model. The 3D datasets were constructed based on the Neuroimaging Informatics Technology Initiative (NIFTI) format, resulting in 114 3D images. These 3D datasets allowed the proposed classification models to be better trained by using the 3D characteristics of the patient’s head, instead of using the less suitable sectional images.
2. A new multi-balancing 3D convolutional neural network (CNN) methodology was proposed to build a 3D classifier model that can work properly with 3D CT scan images and balance itself over the heavy unbalanced multi-classes. Different balancing strategies were evaluated with the proposed models, and it was concluded that the most suitable balancing strategy for the MDCT dataset was a multi-balancing strategy called the synthetic minority oversampling technique with edited nearest neighbor (SMOTE ENN). Moreover, two different 3D CNN classifier models were developed, inspired by the model reported by Zunair et al. (3DSIZ) [11]. In order to perform better with 3D images, the two developed models further performed data augmentation of the cadaver head images, equally normalized the dataset with full volume voxel intensity, and controlled the max polling 3D and dropout layers after multiple experiments.

Intensive experimental studies were conducted, where 18 different experiments were executed with different setups and strategies. The results of these intensive studies were analyzed empirically as well as clinically by a specialized radiologist, in order to assess the validity of the performance of the models. The empirical studies compared the newly developed models, named 3DM1 and 3DM2, with the 3DSIZ baseline model and confirmed that the new models showed an improved performance compared to the baseline model. Moreover, three re-runs of prediction on the test datasets demonstrated that the developed models produced consistent results.

The rest of this paper is organized as follows. In Section 2, the literature of using machine learning (ML) methods in CT diagnosis is presented. In Section 3, the proposed methodology to handle the unbalanced number of ALADA images vs. the above/below-ALADA images when training models are used to predict the dose designation is discussed in detail. In Section 4, the results of the experiments are analyzed, and the clinical expert feedback discussed. In Section 5, the final findings and contribution are discussed, and our conclusions are presented, and future research directions will be recommended.

## 2. Literature Review

In dental implant surgery, MDCT and cone-beam computed tomography (CBCT) are the most common diagnostic imaging modalities used for preoperative surgical planning. The MDCT/CBCT scans use ionizing radiation on patients to depict an anatomical structure within 3D images. Enhancing the resolution of the images requires an increase in the radiation dose [12]. However, a high radiation exposure is not recommended for patients [13]. Numerous DL techniques have been proposed to aid in dental diagnostics [14].

Khanagar et al. [15] reviewed the literature published between the years 2000 and 2020 on AI methods applied in dentistry. In their review, 43 papers were grouped into six groups depending on the dentistry specialty: oral and maxillofacial radiology and diagnostics, orthodontics and dentofacial orthopedics, endodontics, periodontics, oral and maxillofacial surgery, and forensic odontology. The AI methods were implemented on different types of image data: periapical radiographs, dental panoramic radiographs, near-infrared transillumination (TI) images, bitewing radiographs, CT images, CBCT images, lateral cephalometric radiographs, and confocal laser endomicroscopy images. Different DL methods were adopted across publications: artificial neural network (ANN), CNN, probabilistic neural network (PNN), and deep convolutional neural network (DCNN). These methods were reportedly successful in tooth identification, decay detection, dental lesion localization, cephalometric landmark identification, deciding if a tooth needs to be extracted as well as predicting facial swelling after the extraction of teeth. The paper concluded that DL methods can be successful in identifying radiographic findings, diagnosing dental conditions, and planning dental treatment.

Ossowska et al. [16] also reviewed AI methods in dentistry that were reported in 25 papers published between the years 2009 and 2021. The studies included in the review used neural networks to perform tasks related to restorative dentistry, endodontics, orthodontics, dental surgery, and periodontology. The data provided to the neural network models, regardless of the application, were radiographic images. Large-scale applications and methods were described in the studies. However, due to the limited training datasets that were obtained from single sources, the sensitivity of the AI models was an issue that needed to be improved. Hung et al. [17] also reviewed the current development and performance of AI applications in dentomaxillofacial radiology. One application used DL techniques to enhance scanned CBCT images without the need for dosage increase. It was concluded that, even though the reviewed AI models showed promising results, they still had limitations. In particular, the data used to train the AI models were few and collected from the same sources, and many developed models were trained and tested using only confirmed cases. Thus, it is expected that the models are overfitting and cannot be generalized over diverse patients or different scan devices.

A systematic review was conducted by Issa et al. [18] to investigate AI methods used for the specific task of detecting the IAC within CBCT images. The authors concluded that CBCT 3D images allowed practitioners a comprehensive view of the IAC, and that the lack of uniform reporting of the methodology and results affected the quality of the published work.

Several studies have described their methodology for the development of AI applications for the detection of the IAC or other findings within 2D radiographic images. Ekert et al. [19] applied a DCNN to detect apical lesions in dental panoramic radiographs. The proposed 7-layer CNN model was trained over a synthesized dataset of 2001 tooth segments from dental panoramic radiographs and parameterized by a total of 4,299,651 weights. As a result of evaluating the model for detecting six types of tooth sensitivity, it was found that the performance was satisfactory but sensitive, and that the model performed differently with each tooth type. This finding was attributed to the fact that the model was trained on a limited amount of image data. Additionally, Uma Maheswari et al. [20] proposed a new feature-based ML method to detect the IAC within dental panoramic radiographs for pre-diagnostic surgical planning in dental implantology. Image enhancement techniques were adopted including S-CLAHE to enhance the soft features. The proposed method took the shape and textual features of the images as input to detect the regional points in IAC images using a polynomial curve fitting approach. As a result of the empirical study, it was found that the proposed method improved the performance with an accuracy of 96% compared to other traditional ML methods. However, the proposed model works with 2D images and needs textual data to complement the imagery input to be able to perform.

Sukegawa et al. [21] proposed a DL model to analyze the relationship between the mandibular third molar and the IAC in dental panoramic radiographs. The dataset collected included 1279 images of mandibular third molars and the IAC in panoramic radiographs. The reference standard regarding the actual position of the third molar in relation to the IAC was 3D imaging, either CT or magnetic resonance imaging. The dataset was used to train the ResNet50 and ResNet50v2 DL methods, with sharpness-aware minimization (SAM) and stochastic gradient descent as optimizers. The ResNet50V2 model showed an average performance in continuity analysis with 76.6% accuracy, although it outperformed the experts’ diagnosis, which averaged 63% accuracy. On the other hand, the ResNet50V2 model showed a slightly better performance in contact analysis with 86% accuracy. Such results reiterate the effectiveness of using DL in radiographic assessment, but further improvements are still needed.

Kim et al. [22] developed an AI tool to analyze dental panoramic radiographs and predict the occurrence of paraesthesia of the IAC after the extraction of mandibular third molars. The authors concluded that 2D images may negatively contribute to the accuracy of the AI model, and that 3D images may overcome the limitations of the 2D images.

Other studies have described AI methods for the detection of the IAC and other dental structures within 3D CBCT images. Lim et al. [23] explored the use of DL to accurately locate the IAC within the CBCT images. They collected their 3D CBCT images from 138 patients at three hospitals, taken by three different machines. A nnU-Net DL model for segmentation (with active learning) was trained using the images to detect the IAC. The model performance was average due to image noise, the irregular shape of the IAC, and deformation or an unclear image of the IAC. However, it was possible to confirm that DL techniques have a potential to overcome the data difficulties and the model developed needs further improvement.

Lahoud et al. [24] developed a tool that uses feature pyramid network (FPN) to detect teeth within the CBCT images. The authors acquired 314 CBCT images and segmented them manually into the Digital Imaging and COmmunications in Medicine (DICOM) format. After that, 433 DICOM images of individual teeth were selected to train the tool. The authors evaluated their tool against expert segmentations and found that the fully automated tool performance in detecting teeth was “as good as the human operator” with around a 94% accuracy. Moreover, the tool was faster than the experts, with the tool averaging 25 s for completion of the segmentation compared to the experts’ average time of 6 min.

Cui et al. [25] developed an AI system to identify teeth and alveolar bone from CBCT images. They manually labeled 4938 CBCT scans from 15 dental centers and fed the labelled scans into their DL-based system. This study claimed to be the first to combine automated alveolar bone detection and tooth identification. The identification of the two anatomical structures happened simultaneously. The system prepares the images by differentiating between the tooth and no-tooth structures, and sharpening the image contrast for the purpose of identifying alveolar bone. After highlighting the tooth structures, the image is fed into two networks to localize the center and the skeleton of each tooth. At the same time, the sharpened image is processed by a segmentation network to extract the alveolar bone. The accuracy of tooth segmentation was found to be approximately 93%, while the segmentation accuracy for alveolar bone was approximately 94%. Their AI system outperformed the experts with regard to the time taken to complete the task. Furthermore, the authors emphasized that having the AI model perform automatic segmentation of 3D data produced smoother surfaces of the anatomical boundaries compared to the manual segmentation performed by the experts on multiple 2D images.

To facilitate AI processing of the 3D images, Zunair et al. [11] developed a new DL technique that can overcome the computation requirements of 3D images, in addition to leveraging the 3D information. The authors evaluated different uniformizing methods in the 3D image domain to explore sampling a subset of image slices that can construct the desired volume images. The proposed technique was adapted to the CNN method, resulting in a new model called 3DSIZ. The developed model was tested with the lung CT scan domain, and was shown to have improved performance in tuberculosis severity assessment, compared to methods applied with 2D slices and leveraging its metadata. However, the accuracy of the model was only 67.5%, which indicates that the use of DL with 3D images can overcome the current limitations of 2D images, but still needs further improvements in performance.

As such, it can be seen from the review of the literature that the performance of DL methods is related to the data characteristics and is task specific. Additionally, there is a low number of studies reporting on the performance of AI models using 3D scans due to the scarcity of labeled datasets and the struggle of collecting large datasets [14]. In addition, most dental image classification methods reported in the literature were trained using devices with similar characteristics. Thus, such AI methods cannot be generalized to other types of devices or reconstruction techniques or tissues. Furthermore, to the authors’ knowledge, there are no published works that have demonstrated the use of AI to predict the ALADA dose for the identification of the position of the IAC. Therefore, the aim of the present study was to develop an AI tool to analyze MDCT images of cadaveric heads or mandibles acquired with variable devices, reconstruction algorithms, and doses as well as to identify the MDCT ALADA dose for the identification of the IAC. Such a tool could be used across various MDCT devices, and would enable device manufacturers and/or end users to optimize the IAC examination protocols with minimal time and effort compared to existing practices (which require time and labor extensive studies for each newly developed device). Such dose optimization would reduce the potentially hazardous radiation exposure to patients.

## 3. Proposed Methodology

**Figure 1:**
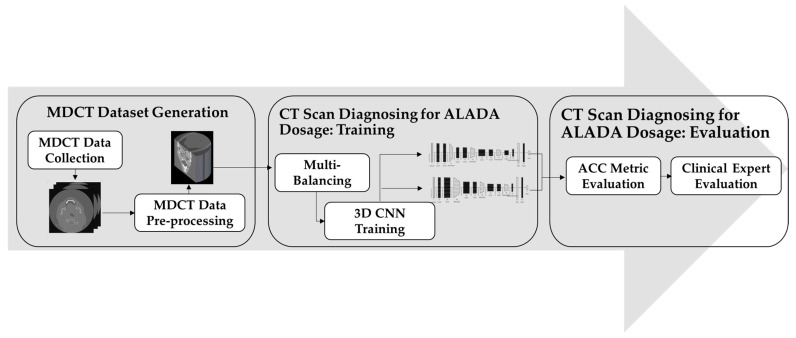
Proposed CT scan identification of the ALADA dosage methodology.

In order to build the CT scan classification model for ALADA dosage, this paper adopted the methodology illustrated in Figure 1. The proposed methodology starts with collecting the MDCT datasets. The datasets used were acquired using two different MDCT scanners with different combinations of reconstruction techniques and radiation doses, as described in previous cadaveric studies [8,9]. The resulting MDCT image datasets were in DICOM format, and each dataset was composed of hundreds of axial sectional 2D images, which individually do not fully reflect the examination characteristics. Thus, the second step was introduced to reconstruct the images in a 3D format.

After the datasets were prepared, the classifier needed to be trained. However, there was a huge imbalance between the number of datasets in each class. As the number of MDCT datasets labelled as ALADA was much lower than the number of datasets labelled as higher or lower than ALADA, a third step was introduced, in which multi-balancing strategies were applied to re-sample the data and generate a new version of the dataset with better distribution. Finally, in the fourth step of the proposed methodology, an improved 3D CNN method was applied to train the model to classify the CT scan images and predict which images were acquired with the ALADA dosage. This paper introduced different 3D CNN architectures and evaluated them scientifically and by clinical experts in order to measure not only the performance of the developed models, but also their impact, as will be explained in the experiments. The following sections will discuss the details of each step and how it was implemented.

### 3.1. Multidetector CT (MDCT) Dataset Generation

A total of 114 MDCT datasets were collected from previous cadaveric studies [8,9] that investigated various combinations of MDCT scanners and reconstruction techniques to determine the ALADA dose for the identification of the position of the IAC for each combination of the scanner and reconstruction technique. The datasets were collected using two MDCT scanners under different combinations of dose/reconstruction techniques. The first MDCT scanner (scanner1) was used to scan three full cadaveric heads using 22 different combinations of the dose and reconstruction technique [9]. Therefore, each combination of dose/reconstruction technique yielded three datasets, for a total of 66 datasets acquired from the first MDCT scanner. The second scanner (scanner2) was used to image four cadaveric mandibles only (bone with attached muscles and tongue), using 12 different combinations of the dose and reconstruction technique [8]. Therefore, each combination of the dose/reconstruction technique yielded four datasets, for a total of 48 datasets acquired from the second MDCT scanner.

#### 3.1.1. DICOM Dataset Characteristics

All of the datasets were exported in DICOM format, and each dataset was a volumetric dataset composed of hundreds of axial sectional images. Using scanner1, the number of sections acquired for each cadaver were as follows:

- Cadaver code #4072: 273 sections;
- Cadaver code #4116: 241 sections;
- Cadaver code #4142: 257 sections.

The total number of image sections for the three cadavers within each combination of dose/reconstruction technique was 771 sections.

Using scanner2, the number of sections acquired for each cadaver was as follows:

- Cadaver code #2089: 249 sections;
- Cadaver code #2120: 226 sections;
- Cadaver code #2140: 234 sections;
- Cadaver code #3128: 236 sections.

The total number of image sections for the four cadavers within each combination of dose/reconstruction technique was 945 sections.

Table 1 outlines the number of image sections and their ALADA designation according to the MDCT scanner, reconstruction technique, and dose. As such, the number of MDCT datasets and sectional images acquired from each MDCT scanner was as follows:

- Scanner1:
  - Higher than ALADA: Nine datasets with 2313 sectional images;
  - ALADA: Six datasets with 1542 sectional images;
  - Lower than ALADA: Fifty-one datasets with 13,107 sectional images.
- Scanner2:
  - Higher than ALADA: Twenty-four datasets with 5670 sectional images;
  - ALADA: Twelve datasets with 2835 sectional images;
  - Lower than ALADA: Twelve datasets with 2835 sectional images.

In total, 4377 sectional images were designated as acquired with an ALADA dose, 7983 sectional images with a higher than ALADA dose, and 15,942 sectional images with a lower than ALADA dose. Using scanner1, the total number of image sections for the three cadavers within each combination of dose/reconstruction technique was 771 sections, while when using scanner2, the total number of image sections for the four cadavers within each combination of dose/reconstruction technique was 945 sections.

#### 3.1.2. MDCT Dataset Preprocessing

The collected datasets were in the DICOM format, and were composed of hundreds of axial sectional images. This format, however, was not designed to facilitate efficient data manipulation and image processing [26]. Since different scanners were used to collect the data with different combinations of reconstruction techniques and doses; the resultant images were varied and the slice thickness and spacing between them were different. Furthermore, when testing the proposed model on the original datasets in the DICOM format, it was found that training on the sectional images was not suitable and many of the 3D characteristics of the patients’ head were lost, thus the model did not perform well. To resolve this issue, the reconstruction of DICOM images into 3D representations was necessary in order to accurately reflect the examination characteristics.

**Figure 2:**
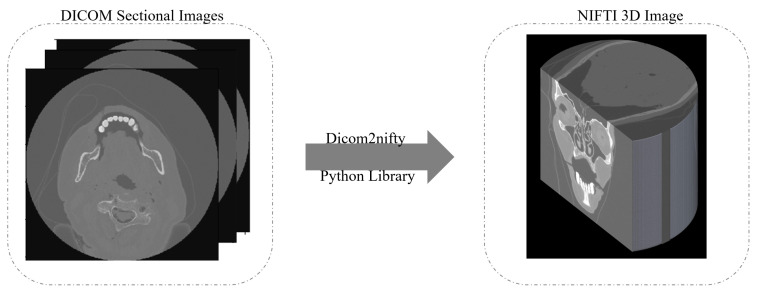
3D image construction to convert DICOM files into 3D NIFTI images.

One of the best known methods to generate a 3D image is to convert the DICOM files into the Neuroimaging Informatics Technology Initiative (NIFTI) format [27]. As illustrated in Figure 2, the 3D reconstruction starts by taking the DICOM sectional images as input slices. Then, using the dicom2nifiti library, the 3D image is generated as a NIFTI image that provides a 3D representation of the whole head. When converting the DICOM files into the NIFTI format, the slices are stacked together into individual volumes, and the volumes are grouped together into their corresponding scan sequence. This makes the data much more compact and easier to process.

#### 3.1.3. NIFTI Dataset Characteristics

After processing the DICOM slices into 3D NIFTI images, the collected datasets were composed of 114 3D images, where 18 3D images were designated as acquired with an ALADA dose, 33 3D images with a higher than ALADA dose, and 63 3D images with a lower than ALADA dose. Table 2 demonstrates the total number of imaged cadavers and their ALADA designation, according to the MDCT scanner, reconstruction technique, and dose. It was clear that there was a high imbalance in this dataset, which might affect how well the classification model would perform with each class, as explained in Section 3.2.

### 3.2. Muti-Balancing 3D Convolutional Neural Network

**Figure 3:**
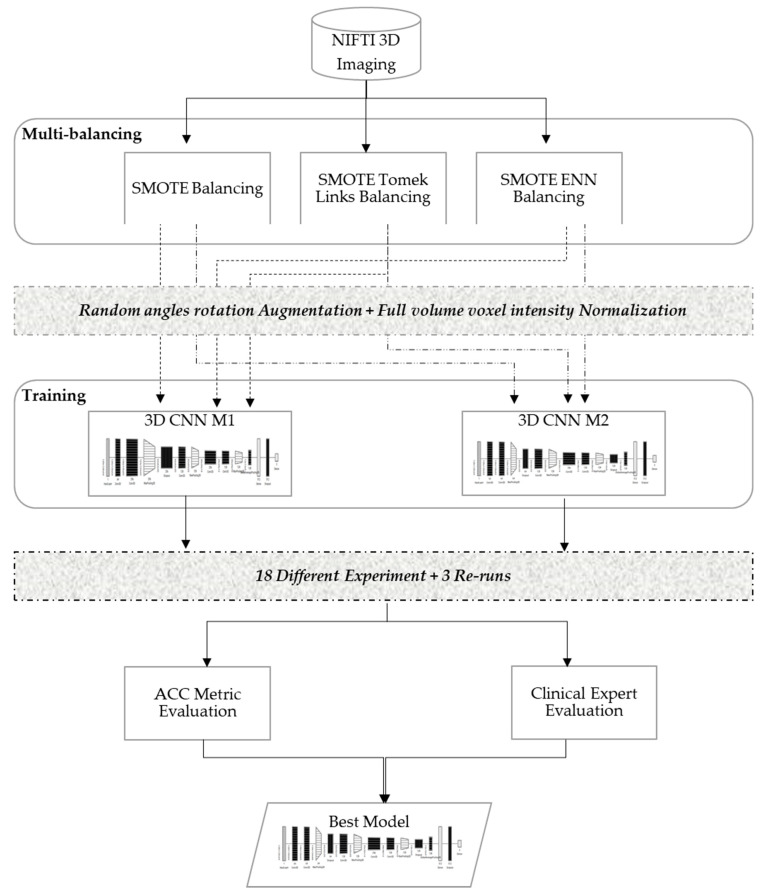
Multi-balancing 3D CNN method.

In order to develop a robust classifier that can work properly with 3D images and balance itself over the heavy unbalanced classes, this study evaluated various dataset-balancing strategies and developed a new 3D CNN modeling pipeline, as illustrated in Figure 3. The proposed method performed data augmentation over the cadaver head images with random angle rotation in the range of −20° to +20°, reserving 70% of the datasets for training, and 30% of the datasets for validation. This section explains the details of the developed multi-balancing strategies in addition to the 3D CNN architectural details used in our method.

#### 3.2.1. Multi-Balancing Strategies

An imbalanced dataset is a property of a dataset where classes are not equally distributed and carry higher noise, resulting in model overfitting [28]. Applying an inappropriate strategy over an unbalanced dataset can be dangerous, especially when it is used for medical purposes, as in the case of this study.

In the literature, researchers have introduced different strategies to re-balance data distribution, in which the training process is adjusted to increase minority class instances while reducing the majority class instances [29]. In particular, the oversampling strategy is one of the well-known data balancing strategies in the literature when dealing with medical images. These strategies have also been extended into multi-balancing strategies, where oversampling and undersampling are combined to balance the data. However, to the authors’ knowledge, these strategies are usually applied over datasets with binary classes, and their performance with multi-class medical images have not yet been investigated. Therefore, this paper evaluated the following three balancing strategies using the standard hyperparameter configuration illustrated in Table 3 and the ACC metric.

- Synthetic minority oversampling technique (SMOTE) [30], which is an oversampling strategy to create synthetic data points for minorities classes.
- SMOTE Tomek Links [28], which first applies SMOTE and then a heuristic under-sampling strategy to remove the borderline data features.
- SMOTE edited nearest neighbor (ENN) [28], which applies SMOTE and then an under-sampling technique that removes the majority of borderline data features identified by K-nearest neighbor.

The key observations noted during the experimental runs were that the models were underfitting and/or overfitting in early stopping batches and yielded a higher noisy prediction output. Underfitting and noisy output were generated when using the imbalanced dataset, and the model was more biased toward most samples (i.e., lower ALADA with 63 samples compared to 33 higher ALADA and just 18 for ALADA), even though random image orientations were employed. However, the prediction output of the model was improved as oversampling and under-sampling strategies were employed on the datasets. Particularly, the best-fit model for the ALADA dose predictions used the SMOTE ENN strategy for dataset balancing. Thus, this multi-balancing strategy was adopted with the proposed 3D CNN models.

#### 3.2.2. 3D CNN Models Architecture

In this paper, two 3D CNN models, Model 3DM1 and Model 3DM2, were proposed to classify the CT scan images and predict the ALADA designation. The 3D CNN models proposed are an extension of 2D CNN, a neural network specifically designed to process spatiotemporal data such as videos or volumetric medical images [31]. Commonly, 3D CNN-based models consist of different layers such as the convolutional neural network, pooling, dropout, and fully connected layers. The design of convolutional layer filters in our models included another dimension that allows for capturing the depth of input data, which helps in the specificity of classification problems. Similarly, pooling layers in the models used to downsample the spatial feature map size, and a complete network of connected layers in the model helped improve the classification convergence for 3D medical images to capture multi-scale features and a better classification performance was observed.

The proposed models, as illustrated in Table 4, were inspired from the model described by Zunair et al. [11], called 3DSIZ. However, unlike the 3DSIZ model intensity, the MDCT datasets were equally normalized with the full volume voxel intensity (−1024 ~ 2000) while keeping the volume depth at 64 × 128 × 128. Moreover, the proposed 3D CNN models had controlled max polling 3D and dropout layers, which were selected after multiple experiments. Our model also implemented a regularization technique using the dropout layer to avoid model overfitting.

The proposed models used the maximum volume voxel intensity, which positively affected the model performance, due to the inclusion of comprehensive cadaveric anatomy and examination characteristics.

## 4. Experimental Results

In order to evaluate the multi-balancing 3D CNN models developed in this study, their performance was measured and compared with the baseline model (3DSIZ). This section will detail the experiment setup and discuss the results from the machine and expert perspectives.

### 4.1. Experiment Setup

In this study, different experiments were orchestrated using several balancing strategies and architectures to evaluate the inferencing of the models. The experiments were conducted in two different pipelines. The first pipeline run was conducted through a full 100 epoch, and the second pipeline run used early stopping methods. After 18 experiments, it was concluded that the best hyperparameters were the ones illustrated in Table 5 and, thus used to compare the performance of the developed models and the benchmark model (3DSIZ).

In the experiments, the developed models (3DM1 and 3DM2) were tested and compared to the benchmark model (3DSIZ), with 70% of the dataset for training and 30% for validation. For each run, the ACC metrics [33] were computed using Equations (1) and (2) respectively. With regard to the loss function, illustrated in Equation (1), the cross-entropy loss function was used after applying categorical one-hot encoding (as 0, 1, and 2) on a given multi-classes dataset to classify the probability of class from 0 to 1. When the probability result is closer to one, it means a higher label probability, while a probability that is close to zero means less probability of that label. In Equation (1), the *p*(*X*) is the probability of the dose prediction for all classes separately and *q*(*X*) is the base-2 log for that event. Alternatively, Equation (2) is the accuracy function that is used to measure the model accuracy by dividing all the correct classifications over the total classifications. In Equation (2), the *TP* is the number of true positives, *TN* is the number of true negatives, *FP* is the number of false positives, and *FN* is the number of false negatives.

(1) $$\ell \left(p,q\right)=-\underset{x}{\sum }p\left(X\right).\log q\left(X\right)$$

(2) $$Accuracy=\frac{TP+TN}{TP+TN+FP+FN}$$

### 4.2. Performance Analysis

The overall performance of the models is shown in Table 6. The proposed models, 3DM1 and 3DM2, improved the performance compared to the baseline model (3DSIZ). Compared to the baseline model, 3DM1 and 3DM2 improved the accuracy by ~5% and ~10%, respectively. Furthermore, the loss with 3DM1 and 3DM2 was reduced by ~0.39 and ~0.41, respectively, when compared with the baseline model. These results indicate that the proposed models significantly improved the performance of the 3D CNN over the unbalanced MDCT dataset. In particular, 3DM2 showed more significant improvement than 3DM2, indicating that its architecture is more suitable.

**Figure 4:**
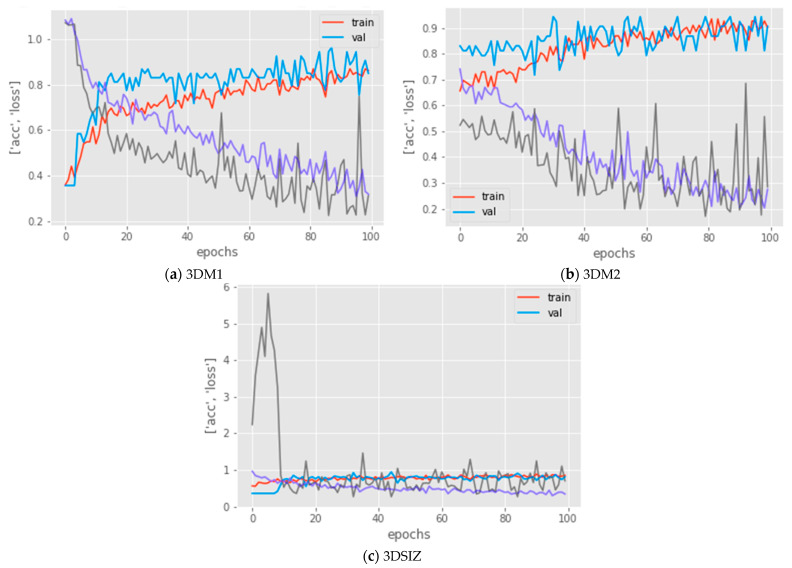
Learning curves that show the accuracy and loss of the training dataset (colored red and gray, respectively) against the accuracy and loss of the validation dataset (colored blue and purple, respectively) for the three models: 3DM1, 3DM2, and 3DSIZ.

In addition to the total performance, an analysis of how the models behave during the evaluation is also important (see Figure 4). This analysis can provide insights into how much the model is underfitting or overfitting and how stable it is, considering the complexity of the data. With 3DM1, as shown in Figure 4a, the training and validation accuracy smoothened out in a normal fashion due to the ENN data balancing strategy, with slightly higher noise due to data augmentation compared to 3DM2, as shown in Figure 4b. The same behavior was also noticed when comparing the loss results of 3DM1, which also confirmed that the model did not overfit or underfit the dataset.

The results of the 3DM2 model, illustrated in Figure 4b, demonstrated a good fit model. The training and validation accuracy smoothened out in a normal fashion, and the noise was lower. Moreover, similar to 3DM1, the loss results of the 3DM2 model also confirmed that the model did not overfit or underfit the dataset.

However, the results of the baseline model (3DSIZ) presented in Figure 4c demonstrated that this model performed poorly. The accuracy of the training and evaluation was almost the same, showing that the 3DSIZ model was underfit, and the loss results were highly unstable, showing that the model was not suitable for the given multi-class dataset.

With regard to the CPU time, all of the experiments were conducted on the cloud with a virtual environment of the NVIDIA RTX A4000 GPU and Linux operating system. After measuring the training time of each model, it was found that 3DM1 and 3DSIZ took 29 min and 1 s to finish, while 3DM2 needed 54 m 11 s to complete. Although 3DM2 took double the time to finish compared to 3DM1 and 3DSIZ, consuming less than an hour for training over 3D images is still a relatively short time compared to the times reported in the literature.

### 4.3. Clinical Expert Analysis

As per the standard practice in health care, clinical experimentations on the best models (3DM1 and 3DM2) were performed with a specialized radiologist. For each model, three re-runs of prediction on the test datasets returned identical results. Table A1 and Table A2, shown in the Appendix A, demonstrate the designation of the dose levels (labels) of the individual image sections within each MDCT dataset by 3DM1 and 3DM2, respectively. The label assigned to the majority of the individual image sections within each MDCT dataset was considered to be the label for the dataset. Erroneous labelling of the majority of the individual images within the dataset was considered as an erroneous label for the entire dataset.

Table 7 demonstrates the total number of MDCT datasets designated as one of the three specific dose labels by the 3DM1 and 3DM2 models compared with the true label. Both models accurately labelled 94% of the MDCT datasets (50/53 datasets). For one of the datasets that was incorrectly labeled by 3DM1, the difference between the correctly labelled and incorrectly labelled individual sections within the dataset was only 5.9%. For two of the datasets incorrectly labeled by 3DM2, the difference between the correctly labelled and incorrectly labelled individual sections within the datasets was only 2.5% and 10.1%, respectively.

### 4.4. Discussion

Dose optimization, or determining the ALADA dose, in CT imaging is needed to avoid any unnecessary increase in the risk of potential harm to patients due to the exposure to ionizing radiation. The dose optimization is especially important in light of the findings of the United Nations Scientific Committee on the Effects of Atomic Radiation Sources and Effects of Ionizing Radiation [1], which reported that, in some countries with a high level of health care, the collective ionizing radiation dose to the population from medical sources was close to, or greater than, the background radiation. The report attributed the increase in collective dose to the increasing availability of CT examinations. Currently, the most effective method for dose optimization is conducted manually, and is labor and time intensive, and the results cannot be generalizable to machines from different manufacturers, different models from the same manufacturer, or even the same model if different software algorithms are used to process the images [8,9,34,35]. Furthermore, the results of dose optimization studies have quickly become obsolete with the development of more advanced CT machines, even by the same manufacturer. As a result, there is a lack of strong scientific evidence identifying the lowest appropriate CT machine specific radiation doses for CT imaging for most diagnostic tasks.

The present study has provided proof-of-concept that AI models, especially 3DM2, can be used for automatic and rapid dose optimization. The developed models described in the present study allowed for the accurate designation of the ALADA dose for identification of the IAC, thus they may help MDCT manufacturers and end users to reduce the radiation exposure to patients by informing their selection of the lowest diagnostic dose possible. The models were applied to the resultant images of the cadavers, and thus independent of the CT device or algorithm. As such, the use of the present models has the potential to be generalized across different CT machines and algorithms.

Furthermore, the technique developed in the present study may potentially be used with other diagnostic tasks, especially related to soft tissue CT imaging, which imparts relatively higher radiation doses. However, this method for developing AI models, which can identify the ALADA dose, still requires manual labor to label the data to train the models. However, once the models are trained successfully to identify the ALADA dose for a specific diagnostic task, the manual technique is no longer needed. The proposed clinical application of the models developed in the present study would be to use the MDCT device and reconstruction technique being tested to acquire multiple examinations of cadavers or tissue-mimicking phantoms by using progressively lower doses. The 3DM2 model may then be applied to the resultant images to identify which images were acquired with the ALADA dose. Any new MDCT device or reconstruction technique could be tested in this manner. The effect of the 6% error rate of the present models could be overcome by testing numerous series of MDCT examinations, so that the correct pattern of labels would be evident, and any isolated deviation from the pattern could be retested using another MDCT examination.

This study utilized the 3D MDCT data to train the 3DM1 and 3DM2 models, and compared the results with those obtained by applying the 3DSIZ model developed by Zunair et al. [11]. The 3DSIZ model was one of the first models to effectively utilize the 3D data of the MDCT datasets to train a CNN model. However, when that model was used to predict which images were acquired with the ALADA dose in the present study’s test data, the 3DSIZ model was less accurate than the 3DM1 and 3DM2 models. The reason for the inferior performance of the previous model may be due to the fact that it was developed for a different diagnostic task, which was the identification of the severity of tuberculosis from lung images, and was trained using soft tissue MDCT images. The present study, on the other hand, was applied to bone images and involved the identification of the fine bony roof of the IAC. As such, the nature of the images used as well as the diagnostic tasks being investigated were different between the two studies.

One of the limitations of the present study was that after balancing the original data by SMOTE ENN, it was not possible to determine the true exact dose and reconstruction technique of each dataset, nor by which MDCT machine the datasets were acquired. It was only possible to identify the datasets as “ALADA”, “above ALADA”, or “below ALADA”. As such, it was not possible to determine whether the models’ errors were associated with any particular reconstruction technique or MDCT machine. Another limitation of the present study is the limited number of MDCT devices and tested datasets. Furthermore, the newly developed models were not tested on CBCT images, which are increasingly replacing MDCT images in dental implant site imaging. The performance of the developed models when applied to CBCT images may conceivably be different than when applied to MDCT images because CBCT images have different physics of image acquisition and image quality profiles compared to MDCT images [36,37]. As such, recommendations for further improvement of the developed models include further training of the models using CBCT images, testing the models on a larger number of CT datasets (both MDCT and CBCT), and relating the performance of the developed models to the exact CT machine and reconstruction algorithm in order to identify any possible causes of the errors. Another research direction would be to evaluate the ensemble learning methods and improve the model accuracy with newly sampled data.

## 5. Conclusions

This paper illustrates the potential use of DL technologies to classify 3D CT scan images and find the optimal ALADA dosage for ionizing radiation. Through the developed 3D MDCT dataset, it was possible to preserve the patients’ head characteristics and introduce scalability by collecting images from different devices that were reconstructed using different methods. The proposed multi-balancing 3D CNN models (3DM1 and 3DM2) were robust classifiers that worked properly with 3D images and balanced themselves over the heavy unbalanced classes. The empirical results, especially of the 3DM2 model, showed a significant improvement in performance when compared to the baseline CNN model. As confirmed by a specialized radiologist, the 3DM2 model can eventually help health care service providers to reduce the MDCT radiation dose levels when investigating the position of the IAC. The methods described in the present study were useful in overcoming the problem of a limited imbalanced training dataset. Further investigation is needed to determine whether the models’ errors were associated with a particular reconstruction technique or MDCT machine. Future research may also explore how federated edge learning could be used with DICOM devices, what kind of privacy controls should be established, and how multi-balancing strategies could play an effective role in generating effective diagnostic predictions.

## Figures and Tables

**Table 1:** Total number of image sections and their ALADA designation (ALADA dose, or higher or lower than the ALADA dose) according to the MDCT scanner, reconstruction technique, and dose. CTDIvol: volume CT dose index; LD: low dose protocol; mGy: milli Grays; FBP: filtered backprojection; ASIR: adaptive statistical iterative reconstruction; MBIR: model based iterative reconstruction; SAFIR: sinogram-affirmed iterative reconstruction.

Dose Protocol (CTDIvol in mGy)	Scanner	Reconstruction Techniques					
		FBP	ASIR 50	ASIR 100	MBIR	SAFIRE 3	SAFIRE 5
Reference (29.4)	Scanner1	771 Higher ALADA	-	771 Higher ALADA	-	-	-
LD1 (4.19)	Scanner1	771 Higher ALADA	771 Lower ALADA	771 ALADA	771 Lower ALADA	-	-
LD2 (2.64)	Scanner1	771 ALADA	771 Lower ALADA	771 Lower ALADA	771 Lower ALADA	-	-
LD3 (0.99)	Scanner1	771 Lower ALADA	771 Lower ALADA	771 Lower ALADA	771 Lower ALADA	-	-
LD4 (0.53)	Scanner1	771 Lower ALADA	771 Lower ALADA	771 Lower ALADA	771 Lower ALADA	-	-
LD5 (0.29)	Scanner1	771 Lower ALADA	771 Lower ALADA	771 Lower ALADA	771 Lower ALADA	-	-
Reference (11.27)	Scanner 2	945 Higher ALADA	-	-	-	945 Higher ALADA	945 Higher ALADA
LD1 (3.09)	Scanner 2	945 Higher ALADA	-	-	-	945 Higher ALADA	945 Higher ALADA
LD2 (1.74)	Scanner 2	945 ALADA	-	-	-	945 ALADA	945 ALADA
LD3 (0.67)	Scanner 2	945 Lower ALADA	-	-	-	945 Lower ALADA	945 Lower ALADA

**Table 2:** Number of imaged cadavers and their ALADA designation (ALADA dose, or higher or lower than the ALADA dose) according to the MDCT scanner, reconstruction technique, and dose. CTDIvol: volume CT dose index; LD: low dose protocol; mGy: milli Grays; FBP: filtered backprojection; ASIR: adaptive statistical iterative reconstruction; MBIR: model based iterative reconstruction; SAFIR: sinogram-affirmed iterative reconstruction.

Dose Protocol (CTDIvol in mGy)	MDCT Scanner	RECONSTRUCTION TECHNIQUE						
		FBP	ASIR 50	ASIR 100	MBIR	SAFIRE 3	SAFIRE 5	TOTAL
Reference (29.4)	Scanner1	3 Higher	-	3 Higher	-	-	-	6
LD1 (4.19)	Scanner1	3 Higher	3 Lower	3 ALADA	3 Lower	-	-	12
LD2 (2.64)	Scanner1	3 ALADA	3 Lower	3 Lower	3 lower	-	-	12
LD3 (0.99)	Scanner1	3 Lower	3 Lower	3 Lower	3 Lower	-	-	12
LD4 (0.53)	Scanner1	3 Lower	3 Lower	3 Lower	3 Lower	-	-	12
LD5 (0.29)	Scanner1	3 Lower	3 Lower	3 Lower	3 Lower	-	-	12
Reference (11.27)	Scanner2	4 Higher	-	-	-	4 Higher	4 Higher	12
LD1 (3.09)	Scanner2	4 Higher	-	-	-	4 Higher	4 Higher	12
LD2 (1.74)	Scanner2	4 ALADA	-	-	-	4 ALADA	4 ALADA	12
LD3 (0.67)	Scanner2	4 Lower	-	-	-	4 Lower	4 Lower	12
Total	--	34	15	18	15	16	16	114

**Table 3:** Configuration used in the balancing strategy experiments with full epoch and early stopping runs for 6 runs.

Hyperparameters	Value	Number of Training Sample	Number of Validation Sample
architecture	Conv3D	SMOTE: 132 SMOTE Tomek Links: 130 SMOTE ENN: 121	SMOTE: 57 SMOTE Tomek Links: 57 SMOTE ENN: 53
batch_size	2		
decay_rate	0.96		
decay_steps	100,000		
epochs	100 or early stopping		
learning_rate	0.0001		
loss_function	categorical_crossentropy		
patience	15		
staircase	True		
validation_steps	5		

**Table 4:** Architectures of the 3D convolutional neural network Models 3DM1 and 3DM2, visualized using the tool by A. Bäuerle et al. [32].

Model 3DM1	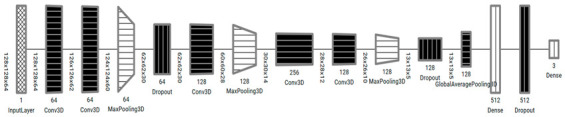
Model 3DM2	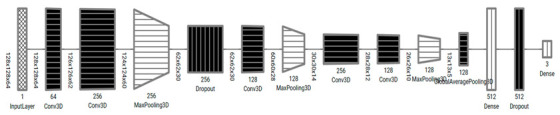

**Table 5:** Standard hyperparameter configuration used in all experiments.

Hyperparameters	Value
batch_size	2
decay_rate	0.96
decay_steps	100,000
learning_rate	0.0001
epochs	100
loss_function	categorical_crossentropy
patience	15
staircase	True
validation_steps	5

**Table 6:** Model performance: ACC metric results.

Model	Balancing Strategy	Accuracy	Loss
3DSIZ	SMOTE ENN	0.811	0.701
3DM1	SMOTE ENN	0.849	0.314
3DM2	SMOTE ENN	0.906	0.284

**Table 7:** The confusion matrix for the prediction of 3DM1 and 3DM2 that shows the number of data objects that were correctly labeled.

True Label	3DM1 Predicted Label			3DM2 Predicted Label		
	Lower ALADA	ALADA	Higher ALADA	Lower ALADA	ALADA	Higher ALADA
Higher ALADA	0	0	15	0	0	15
ALADA	2	16	1 *	1	17	1 **
Lower ALADA	19	0	0	18	1 ***	0

* 5.9% difference only in the number of individual images identified as higher ALADA and ALADA. ** 2.5% difference only in the number of individual images identified as higher ALADA and ALADA. *** 10.1% difference only in the number of individual images identified as ALADA and lower than ALADA.

## Data Availability

Data are unavailable due to privacy or ethical restrictions.

## Appendix A

**Table A1:** Model 3DM1′s designation of the dose levels (labels) of the individual image sections within the test MDCT dataset folders. ALADA: as low as diagnostically acceptable.

Test Folder Number	True Folder Label	Model’s Label of Individual Images within the Folder (%)		
		Higher than ALADA	Lower than ALADA	ALADA
1	Lower than ALADA	1.36	60.42	38.21
2	Higher than ALADA	99.28	0	0.71
3	ALADA	0.1	73.08 *	26.83
4	Lower than ALADA	1.94	68.58	29.49
5	Lower than ALADA	0.02	87.78	12.2
6	Higher than ALADA	61.03	0	38.97
7	Higher than ALADA	77.92	1.08	21.01
8	ALADA	4.97	33.53	61.5
9	Lower than ALADA	3.58	61	35.42
10	ALADA	2.17	61.7 *	36.13
11	Lower than ALADA	1.19	57.99	40.82
12	ALADA	13.17	0	86.83
13	Higher than ALADA	98.51	0	1.49
14	ALADA	0.8	49.02	50.18
15	Lower than ALADA	0.11	74.56	25.33
16	Higher than ALADA	84.03	0	15.97
17	ALADA	1.98	0	98.02
18	Higher than ALADA	99.2	0	0.8
19	Lower than ALADA	0	95.3	4.7
20	Higher than ALADA	97.58	0.04	2.38
21	Lower than ALADA	0.15	70.75	29.11
22	Lower than ALADA	0.01	89.44	10.55
23	Higher than ALADA	99.28	0	0.72
24	Lower than ALADA	1.57	68.9	29.54
25	Higher than ALADA	88.78	0	11.22
26	Lower than ALADA	3.34	94.74	1.92
27	Higher than ALADA	97.98	0.01	2.02
28	Higher than ALADA	98.84	0	1.16
29	Higher than ALADA	99.12	0	0.88
30	Lower than ALADA	2.55	95.79	1.66
31	ALADA	1.19	0	98.81
32	ALADA	7.57	4.42	88
33	Lower than ALADA	0.68	59.42	39.9
34	Higher than ALADA	99.26	0	0.74
35	ALADA	12.66	0	87.34
36	Higher than ALADA	79.1	0.06	20.84
37	Lower than ALADA	2.37	51.96	45.66
38	ALADA	52.95 *	0.02	47.02
39	Lower than ALADA	0.06	83.4	16.54
40	ALADA	6.22	15.55	78.23
41	Higher than ALADA	75.02	0.83	24.15
42	ALADA	10.03	0	89.97
43	Lower than ALADA	0	97.45	2.55
44	Lower than ALADA	5.05	93.23	1.72
45	ALADA	4.13	0	95.87
46	ALADA	1.81	0	98.19
47	Lower than ALADA	0.01	90.08	9.92
48	ALADA	1.21	0	98.79
49	ALADA	1.62	0	98.38
50	Lower than ALADA	0.1	78.81	21.09
51	ALADA	2.54	0.02	97.43
52	ALADA	0.77	0	99.23
53	ALADA	7.27	0	92.73

* Erroneous labelling of the majority of the individual images within the dataset was considered as an erroneous label for the entire dataset.

**Table A2:** Model 3DM2′s designation of the dose levels (labels) of the individual image sections within the test MDCT dataset folders. ALADA: as low as diagnostically acceptable.

Test Folder Number	True Folder Label	Model’s Label of Individual Images within the Folder (%)		
		Higher than ALADA	Lower than ALADA	ALADA
1	Lower than ALADA	5.25	70.55	24.2
2	Higher than ALADA	99.73	0.01	0.27
3	ALADA	0.18	29.21	70.6
4	Lower than ALADA	8.48	72.09	19.43
5	Lower than ALADA	2.59	80.7	16.71
6	Higher than ALADA	88.33	0	11.67
7	Higher than ALADA	96.5	0.18	3.33
8	ALADA	4.56	28.66	66.78
9	Lower than ALADA	7.64	65.45	26.91
10	ALADA	7.55	70.33 *	22.12
11	Lower than ALADA	6.33	67.33	26.34
12	ALADA	5.34	0	94.66
13	Higher than ALADA	98.62	0	1.37
14	ALADA	2.31	9.77	87.92
15	Lower than ALADA	1.26	81.25	17.49
16	Higher than ALADA	79.48	0	20.52
17	ALADA	0	2.69	97.31
18	Higher than ALADA	99.06	0	0.94
19	Lower than ALADA	0	97.18	2.82
20	Higher than ALADA	99.55	0	0.44
21	Lower than ALADA	1.33	74.19	24.48
22	Lower than ALADA	0.02	91.56	8.42
23	Higher than ALADA	99.11	0	0.89
24	Lower than ALADA	7.25	72.22	20.54
25	Higher than ALADA	87.61	0	12.39
26	Lower than ALADA	4.9	92.83	2.26
27	Higher than ALADA	99.42	0	0.57
28	Higher than ALADA	99.51	0	0.49
29	Higher than ALADA	99.51	0	0.49
30	Lower than ALADA	3.28	95.09	1.64
31	ALADA	0.2	0	99.8
32	ALADA	8.25	0.1	91.65
33	Lower than ALADA	1.84	44.02	54.14 *
34	Higher than ALADA	98.53	0	1.47
35	ALADA	5.79	0	94.21
36	Higher than ALADA	88.32	0	11.68
37	Lower than ALADA	5.65	68.32	26.03
38	ALADA	51.25 *	0	48.75
39	Lower than ALADA	0.27	86.16	13.57
40	ALADA	4.36	4.73	90.91
41	Higher than ALADA	97.33	0.14	2.53
42	ALADA	4.05	0.01	95.94
43	Lower than ALADA	0	98.18	1.82
44	Lower than ALADA	7.29	90.1	2.61
45	ALADA	1.26	0	98.74
46	ALADA	0.01	0.92	99.07
47	Lower than ALADA	0	93.28	6.72
48	ALADA	0.17	0	99.82
49	ALADA	0.48	0.01	99.51
50	Lower than ALADA	0.08	65.12	34.8
51	ALADA	0.48	0.01	99.51
52	ALADA	0.01	0.07	99.92
53	ALADA	1.62	0	98.38

* Erroneous labelling of the majority of the individual images within the dataset was considered as an erroneous label for the entire dataset.
